# 336. A 6-Sigma Approach for Follow-up Evaluation of Outpatient Parenteral Antimicrobial Therapy (Project SAFE-OPAT) To Improve Documentation and Post-Discharge Care

**DOI:** 10.1093/ofid/ofad500.407

**Published:** 2023-11-27

**Authors:** Vishakh C Keri, Sajjad Ali, Ahmed Elattma, Lea M Monday

**Affiliations:** Wayne State University, Detroit, Michigan; Wayne State University, Detroit, Michigan; Wayne State University, Detroit, Michigan; Wayne state University School of Medicine, Detroit, Michigan

## Abstract

**Background:**

Outpatient Parenteral Antimicrobial Therapy (OPAT) is challenging to implement safely and difficult for infectious diseases (ID) physicians to monitor in centers without a dedicated OPAT staff. We utilized a 6-Sigma framework to evaluate our OPAT process and define opportunities for improvement.

**Methods:**

In a retrospective cohort study, we screened 5 months of ID consult data for patients who left on OPAT from an urban hospital system (Fig 1). Primary outcome was incidence of adverse event (AE), a composite of either emergency department (ED) visit or all-cause 30-day readmission. Clinical characteristics and completeness of ID documentation were compared between patients with and without an AE. Complete documentation included antibiotic dose, duration, stop date, and a scheduled ID appointment. We simultaneously conducted a 6-sigma analysis involving stakeholders (physicians, case managers, nurses) in focus groups, to generate a process map, Ishikawa diagram, 5-Why’s analysis, and identify heterogeneity in our OPAT process (Fig 2).

**Figure:**
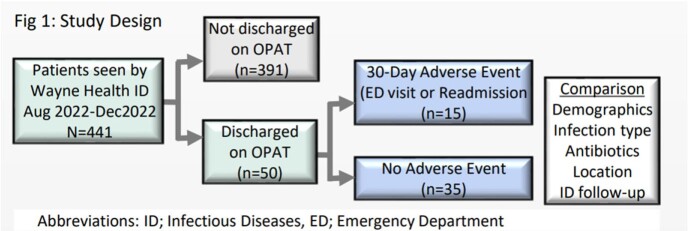

**Figure:**
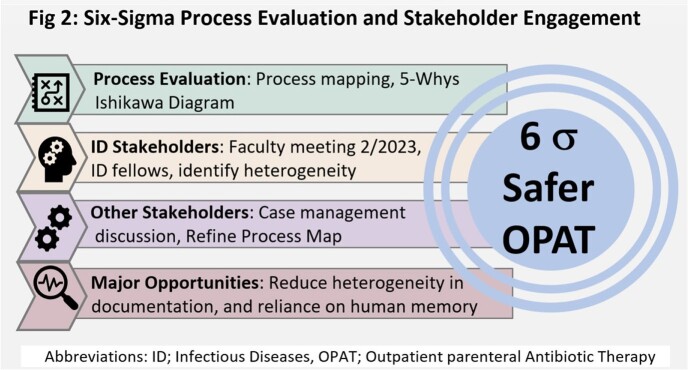

**Results:**

Fifty of 441 patients (11.3%) were discharged on OPAT and incidence AE was 30%. Neither type of infection (Fig 3), nor demographics, clinical characteristics, or discharge location differed between groups (Table 1). Only half (50%) of the patients had complete documentation. Median time from discharge to clinic was 21 days, however, AE’s occurred in a median time of 12 days. Patients without an AE were more likely to have been seen in the clinic post-discharge (51% versus 20%, p=0.039). ID clinic appointments were made for 60% of patients, with a show rate of 63%. (Fig 4). Two additional unscheduled patients initiated their own visit (Fig 4). The 6-Sigma analysis identified process heterogeneity at discharge location and over-reliance on human memory for complete documentation. Interestingly, focus groups revealed numerous assumptions not supported by the objective data (Fig 5 in conclusion).

**Figure:**
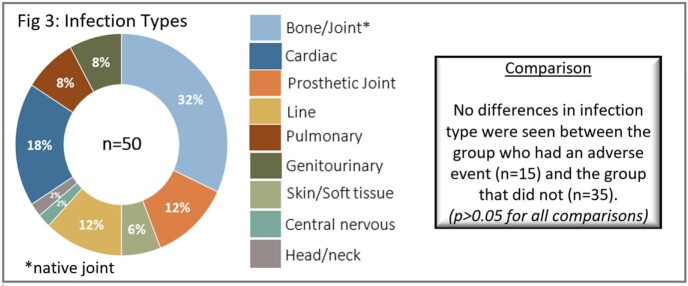

**Figure:**
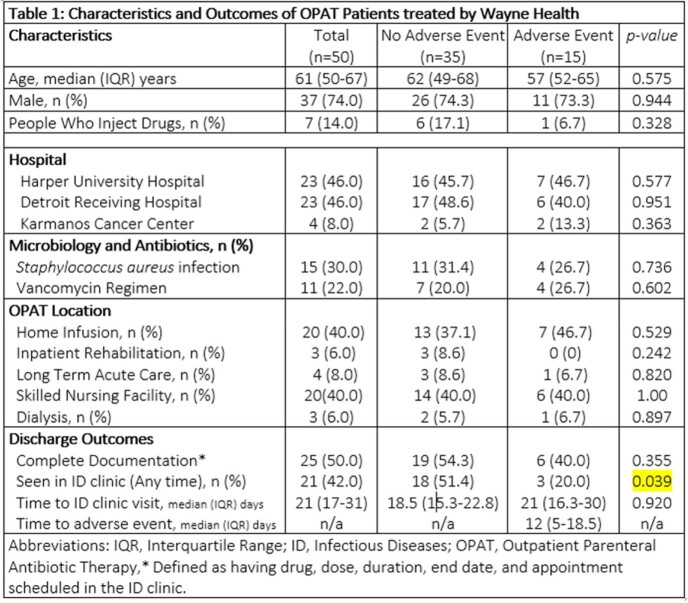

**Figure:**
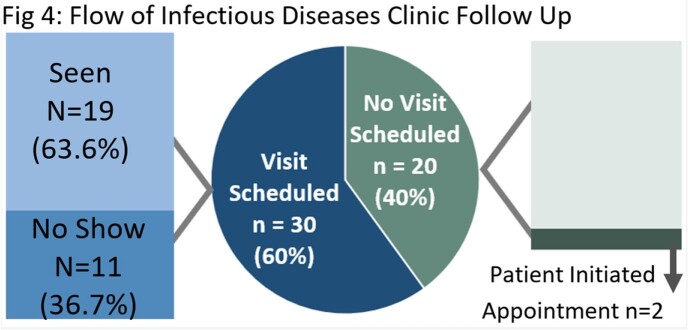

**Conclusion:**

Almost 1 in 3 patients leaving on OPAT experienced an adverse event. A 6 Sigma analysis identified heterogeneity in our process and incorrect assumptions among stakeholders (Fig 5). Next steps should focus on improving ID documentation and ensuring all patients leaving on OPAT have an ID clinic visit scheduled within 14 days after discharge.

**Figure:**
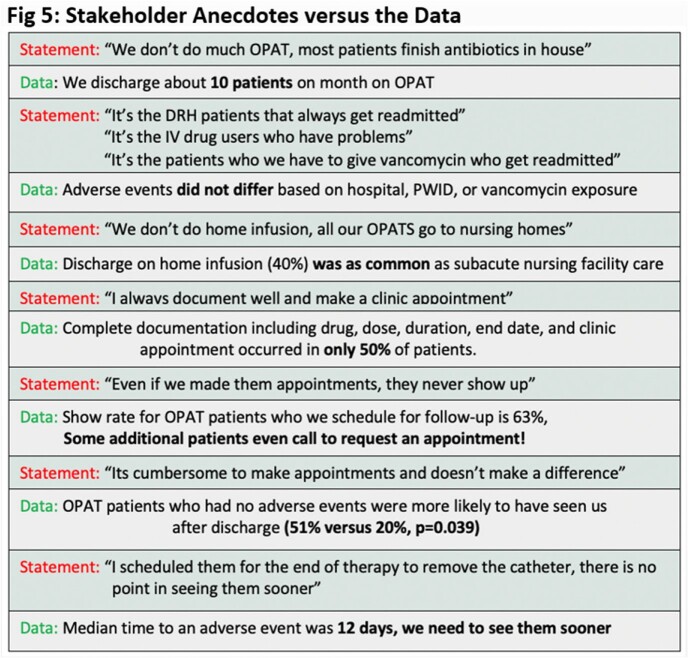

**Disclosures:**

**All Authors**: No reported disclosures

